# Why Numbers Are Embodied Concepts

**DOI:** 10.3389/fpsyg.2017.02347

**Published:** 2018-01-15

**Authors:** Martin H. Fischer

**Affiliations:** Cognitive Science, University of Potsdam, Potsdam, Germany

**Keywords:** arithmetic, numerical cognition, number concepts, embodied cognition, philosophy of science

Number concepts are often thought to be abstractions, for example because the numerosity of sets (e.g., their “three-ness”) is a feature apparently dissociated from the sensory experiences with specific set members, such as their size, shape, or color. In other words, quantity-specific experiences seem to vary arbitrarily when we enumerate three apples, three cars, three people, or three fingers. Hence, Frege (1884) and other logically-minded philosophers considered positive integers as ideal cognitive constructions for enumerative mental operations, removed from contextual constraints, yet preserving precision and generalizing across situations (e.g., arithmetic operations).

Yet, upon closer consideration several sensory and also motor features systematically co-occur with each enumeration we perform; this co-occurrence establishes experiential patterns through which number concepts become embodied as part of their acquisition history (cf. Fischer and Brugger, 2011). I describe here several such systematic co-occurrences and cite supporting evidence. This psycho-logical view of number is not in conflict with but extends purely logical considerations of number concepts as foundations of formal arithmetic, as proposed by Frege (1884).

To-be enumerated objects are usually all simultaneously available to us and thus, by physical necessity, distributed across space because two objects cannot occupy the same place at the same time. Therefore, more objects take up more space and enumerating them invites spatially distributed and temporally extended behaviors; these are sensory cues to number. The systematic directionality of counting behaviors furthermore establishes spatial-numerical associations which, in turn, can be detected with chronometric methods and through behavioral biases (see Fischer and Shaki, 2014; Winter et al., 2015, for reviews).

We know that set members should be aligned or grouped in space to reduce spatial memory load when counting them. We apply verbal sensory-motor routines to establish one-to-one correspondences between objects and number names until each object (or group) was referenced once and the last number name establishes set size or cardinality (e.g., Gelman and Gallistel, 1978). Without such direct referencing of objects through pointing, our eyes and fingers are the universal means of associating body postures (i.e., spatial, visual, kinesthetic, and proprioceptive signals) with number names (Fischer, 2003a; Di Luca and Pesenti, 2011). As a consequence, eye position predicts numerical thoughts (Loetscher et al., 2010), tactile finger stimulation primes number processing and perceiving numbers in turn modulates visual-spatial (Fischer et al., 2003) as well as tactile sensitivity (Tschentscher et al., 2012; Sixtus et al., 2017; Sixtus et al., in revision). Even when overt finger movements are avoided, we spontaneously generate repetitive upper-body movements to enrich our counting with sensory-motor feedback (Carlson et al., 2007).

Habitually, people raised in Western cultures point at horizontally distributed objects left-to-right and thereby associate increasingly larger number names with increasingly more right-sided actions (Opfer and Furlong, 2011; Shaki et al., 2012). The origin of this cultural bias can be traced to observational learning at pre-school age (Göbel et al., 2018) but might have evolutionary origins (Rugani et al., 2015). In other words, the ubiquitous spatial-numerical association of response codes *(SNARC) effect* results from preferred sensory-motor habits.

Two other signature effects of numerical cognition may also be embodied in origin and not only epiphenomenally so. First, when deciding which of two sets is physically larger performance is governed by Weber's law where the just-noticeable difference between them increases with set size. Moreover, we heuristically expect the larger set to also be the more numerous. This pattern is preserved when we distinguish symbolic quantities (i.e., discriminating two digits' meanings). This so-called *numerical distance effect* (Moyer and Landauer, 1967) suggests that we obligatorily recur to the concrete sensory and motor experiences present when these concepts were acquired.

And secondly, when gathering objects, we extend our sensory-motor experiences from the audio-visual and motor to the haptic domain. As a result, wider grasp apertures prime larger numbers (Andres et al., 2004) and number magnitude in turn biases ongoing visuo-motor control (Fischer, 2003b). Holding object sets also lets us experience positive correlations of numerosity and weight. Thus, systematic multi-modal sensory-motor experiences accompany the use of natural number concepts and pose scaled processing challenges for the cognitive system. This is the embodied foundation of the *numerical size effect*, i.e., the systematic increase in processing costs associated with larger numerosities, capturing most everyday experiences, such as managing to juggle 3 but not 4 balls (Fischer, 2017).

To add things up, three cardinal signatures of numerical cognition, the SNARC effect, the distance effect, and the size effect, might be grounded in sensory-motor experiences and in this sense embodied (for a terminological distinction between “grounded” and “embodied” numerical processing, see Fischer, 2012). It is therefore not surprising that we find cross-domain priming in a wide range of tasks whenever people think quantitatively, be they temporal, spatial, or conceptual (Casasanto and Boroditsky, 2008; Scheepers and Sturt, 2014; Walsh, 2015). These associations extend beyond the positive integers or their manipulation in mental arithmetic (e.g., Werner and Raab, 2014) and even shape how we think about negative numbers that cannot be experienced as sensory quantities. An initial report (Fischer, 2003b) associated negative numbers with left-sided space and also showed a size effect (while controlling the distance effect). The finding generated some controversy (reviewed in Mende et al., 2017) but was confirmed when the assessment removed potential biases from spatially distributed stimulus presentation or response recording (Fischer and Shaki, 2017). Our habitual experience with spatially organized magnitudes thus replaces the lack of sensory experience with negative numbers to generate predictable sensory-motor associations.

In conclusion, number concepts, although often used in a context-free and seemingly abstract manner (Frege, 1884), always carry sensory-motor connotations. This correlative experience is used for prediction not abstraction—in other words, we apply concrete experiences gathered within a knowledge domain (the source) to generate predictions that enrich seemingly “abstract” conceptual knowledge (the target domain). Thus, it is only through the embodied lens that we can appreciate the full nature of number knowledge and devise appropriate methods for effective training and rehabilitation of numerical cognition.

## Author contributions

MF: conceived of and wrote this comment and is solely responsible for the content.

### Conflict of interest statement

The author declares that the research was conducted in the absence of any commercial or financial relationships that could be construed as a potential conflict of interest.
